# Impact of robotic platform transition on short term outcomes in colorectal cancer surgeries

**DOI:** 10.1007/s11701-026-03855-y

**Published:** 2026-08-29

**Authors:** Rebeca Hara Nahime, Paulo Roberto Stevanato Filho, Tiago Santoro Bezerra, Tomás Mansur Duarte Miranda Marques, Renata Mayumi Takahashi, Bruna Elisa Catin Kupper, Wilson Toshihiko Nakagawa, Ademar Lopes, Samuel Aguiar

**Affiliations:** Colorectal Cancer Reference Center, A.C. Camargo Cancer Center, São Paulo, SP Brazil

**Keywords:** Robotic surgical procedures, Learning curve, CUSUM, Colorectal neoplasms, Treatment outcome, da Vinci system

## Abstract

Robotic colorectal surgery is increasingly complex, expanding from pelvic to multiquadrant procedures. Reports on the impact of institutional robotic platform transition remain limited. This study evaluated the challenges and effects of transitioning from the da Vinci Si (dV Si) to the da Vinci X/Xi (dV X/Xi) platform. A prospective observational cohort with historical comparison describing an institutional experience that included consecutive elective robotic colorectal cancer resections performed in 2023 (dV Si) and 2024 (dV X/Xi). The primary endpoint was the learning curve for console time. Secondary outcomes included total operative time, port placement time, length of stay (LOS), ileus, anastomotic leakage, severe complications (Clavien–Dindo ≥ 3), reoperation, and 30-day mortality. An institutional learning curve for the surgical team was constructed and evaluated using the cumulative sum (CUSUM) test. A total of 121 procedures were analyzed (dV Si: *n* = 49; dV X/Xi: *n* = 72). Procedural profile shifted significantly (*p* = 0.002), with increased multiquadrant resections in the dV X/Xi group. Console time was longer in dV X/Xi group (*p* = 0.05), while total operative time and LOS were similar in the two groups. Postoperative outcomes were comparable. Parenteral nutrition was less frequently required in the dV X/Xi group (*p* = 0.02). CUSUM analysis showed a console learning plateau at 48 cases.We describe a real-world experience of robotic platform transition, demonstrating that it enabled an expanded range of procedures without compromising short-term outcomes.

## Introduction

Colorectal cancer is the third most commonly diagnosed malignancy and the second leading cause of cancer-related mortality worldwide [1]. In Brazil, the estimated number of new colorectal cancer cases for the 2026–2028 triennium is 53,810 per year, corresponding to an estimated risk of 25.11 cases per 100 thousand inhabitants [2].

Robotic surgery has been progressively adopted as an alternative to laparoscopic and open colorectal surgery over the last few decades [3]. Minimally invasive surgery has demonstrated improved short-term outcomes compared to open surgery, such as less postoperative pain, less blood loss, and enhanced postoperative recovery [4, 5].

Furthermore, robotic technique allows tridimensional vision, steady camera views, flexible robotic arms granting precise dissection and access to confined spaces like the pelvis. The ROLARR trial evaluated robotic versus laparoscopic rectal surgery and suggested potential advantages in selected subgroups [6]. More recently, the REAL trial demonstrated improved oncologic outcomes in robotic rectal surgery [7].

Transitioning a robotic platform unfolds as innovation floods the market, leading to the necessity of technical adjustment in the hospital staff, surgical team and nurses, for the use of the new technology. This adaptation is paramount and can sometimes be troublesome until reaching full team and equipment rapport to maintain surgical timing and adequate outcomes [8].

For eleven years, our institution, A.C. Camargo Cancer Center, has utilized the robotic platform da Vinci Si (dV Si). However, with the discontinuation of the platform by the provider, the technological park was updated to the da Vinci X and Xi (dV X/Xi), leading to the need of team training for the use of the new robots.

Regarding the implementation challenges presented after the arrival of the new robotic platform, we aim to describe the implantation process and technical difficulties encountered. The primary outcome was the learning curve for console time. Secondary outcomes were total operative time, port placement time and postoperative outcomes such as hospital stay, postoperative complications, anastomotic leakage, ileus, reoperation, readmission and 30-day mortality.

## Materials and methods

We conducted an observational, single-center, longitudinal, prospective cohort study compared to a historical control. Consecutive oncologic robotic colectomies and anterior resections performed in 2023 on the da Vinci Si robot (Intuitive Surgical Inc, Sunnyvale, CA), compared to surgeries performed in 2024 on the da Vinci X and Xi robots were included. All procedures were conducted at A.C. Camargo Cancer Center, a quaternary referral center dedicated to oncology care, research, and education, which has maintained a well-established robotic surgery program since 2013, initially utilizing the da Vinci Si platform and transitioning in 2024 to the da Vinci X and Xi platforms.

Data was collected prospectively and extracted from the Early Recovery Protocol database maintained at our institution. This database gathers information on diagnostic, preoperative, intraoperative, and postoperative outcomes, allowing periodic analyses of performance indicators. The study was approved by the A.C. Camargo Cancer Center Institutional Review Board (IRB).

The following surgeries were included: right colectomy (RC), right extended colectomy (REC), left colectomy (LC), total colectomy (TC), sigmoidectomy (S), high and low anterior resection (HAR) (LAR), abdominoperineal resection (APR), and Hartmann procedure (HP). All surgeries were elective. Prior to surgery, patients received mechanical bowel preparation with 1000 ml rectal glycerin and oral antibiotics. As a routine, a protective loop ileostomy was fashioned if LAR was performed.

We evaluated collectively the institutional performance of five oncological colorectal surgeons in the same reference center, in the same period; they were all proficient in robot-assisted surgery. On the arrival of the new robotic platform, the training was a half- period in service to showcase the new features for all senior surgeons. No hands-on training was offered.

Data included patients’ characteristics, diagnosis information, tumor staging, preoperative treatment, type of surgery, robotic platform utilized and type of anastomosis. Intraoperative outcomes were total operative time (from first incision to wound closure), console time and port placement time (from first incision to patient positioning and robot docking).

Regarding neoadjuvant treatment we evaluated if there was no neoadjuvant treatment needed or the need for long course radiochemotherapy (LCCRT) without total neoadjuvant treatment (TNT), LCCRT with TNT- OPRA protocol [9], short course radiochemotherapy (SCRT) without total neoadjuvant treatment (TNT), short course radiochemotherapy (SCRT) with total neoadjuvant treatment (TNT)- RAPIDO protocol [10], LCCRT with TNT and adjuvance with FOLFIRINOX- PRODIGE protocol [11], only TNT- PROSPECT protocol [12], immunotherapy protocols and others [13].

The type of anastomosis was also considered: intracorporeal mechanic anastomosis, robotic intracorporeal single stapled (RISS) [14, 15], transanal transection and single stapled anastomosis (TTSS) [16, 17], double stapled, intersphincteric and end stoma (no anastomosis).

Postoperative complications were classified as severe if Clavien Dindo > = 3, with invasive reintervention defined as the need for surgical, endoscopic, or radiological treatment [18]. Anastomotic leakage was defined as leakage confirmed through imaging or endoscopic exam, faecaloid drain aspect or intraoperative identification. Postoperative ileus was defined as 2 or more nausea or vomiting episodes in 24 h, intolerance to oral intake longer than 24 h, abdominal distension with radiologic confirmation during or after the 4th postoperative day (POD) without spontaneous resolution or need for nasogastric (NG) tube. The incidence of 30-day complications, reoperation and mortality were described.

Data was collected and recorded anonymously on the REDCap platform and exported to SPSS software for statistical analysis. Initially, simple frequencies of variables and outcomes were calculated. Associations between variables and outcomes were assessed using the chi-square or Mann–Whitney test. Associations for categorical variables and comparisons between means of quantitative variables were performed using ANOVA.

A cumulative sum (CUSUM) chart is the running cumulative sum of differences between individual values and the target, demonstrated in a graphical representation of a trend in the outcome of a series of consecutive procedures. In our study, the target is the average of all data points. The inflection point of the CUSUM curve, identified by a change in slope, was considered the transition point from the learning phase to proficiency. This method was applied for our learning curve analysis, and it shows the temporal relationship between the surgical team mastery of a task and the chronological number of cases performed. We used the CUSUM analysis to evaluate surgical performance and define the learning curve of one year of consecutive colorectal robot-assisted resections on each robotic platform [19–21].

## Results

Our patient cohort included a total of 121 colorectal surgeries, 49 (40.4%) surgeries performed in 2023 on dV Si platform and 72 (59.5%) in 2024 on the dV X/ Xi platform. Most of the patients were male 71.4% vs. 73.6%, mean age was 62 (28–86) vs. 56.5 (28–86) on Si and X/Xi, respectively. Mean BMI on both platforms was overweight, and we had American Society of Anesthesiologists (ASA) grade 2 in dV Si 65.3% vs. dV X/Xi 76.4%. Nearly all patients (95–100%) had prehabilitation which included immunonutrition and formal outpatient consults with anesthesiologist, nutritionist, physiotherapist and nurses.

Regarding tumor staging, most of the patients had localized disease: 44 (89.8%) patients operated on dV Si and 68 (94.4%) on dV X/Xi. Lesion location was rectal in 93.9% of Si procedures vs. 61.2% of X/ Xi procedures. Neoadjuvant treatment was omitted in 74(61.2%) patients and when indicated, the most commonly used was the OPRA protocol in 26 (21.5%) patients. Patient demographics and perioperative data are summarized in Table 1.

**Table 1:** Demographics and perioperative characteristics of patient cohort (*n* = 121)

Parameter	dV Si	dV X/Xi
Gender		
Male	35 (71.4%)	53 (73.6%)
Female	14 (28.6%)	19 (26.4%)
Age (years) (median [IQR])	62 (28–86)	56.5 (28–86)
BMI (kg/m²) (median [IQR])	26.86 (18.87–51.3)	28 (18–50)
ASA grading		
Grade 1	7 (14.3%)	6 (8.3%)
Grade 2	32 (65.3%)	55 (76.4%)
Grade 3	10 (20.4%)	11 (15.3%)
Clinical staging (cM)		
cM0	44 (89.8%)	68 (94.4%)
cM1	5 (10.2%)	4 (5.6%)
Tumor location		
Upper/mid rectum	29 (59.2%)	22 (30.6%)
Low rectum	17 (34.7%)	22 (30.6%)
Right colon	0	10 (13.9%)
Transverse colon	0	5 (6.9%)
Left colon	2 (4.1%)	1 (1.4%)
Sigmoid	1 (2.0%)	12 (16.7%)
Neoadjuvant treatment		
None	22 (44.9%)	52 (72.2%)
LCCRT	5 (10.2%)	3 (4.2%)
RAPIDO protocol	6 (12.2%)	0
OPRA protocol	12 (24.5%)	14 (19.4%)
PRODIGE protocol	2 (4.1%)	1 (1.4%)
PROSPECT protocol	2 (4.1%)	0
Other	0	2 (2.8%)

BMI body mass index, ASA American Society of Anesthesiologists

Comparison of patient demographics and operative parameters according to robotic platform are summarized in Table 2. We had interesting results on the type of surgery performed in 2024 with an increased range of procedures including 9 (12.5%) RC, 6 (8.3%) REC, 2 (2.8%) TC and 10 (13.9%) S. On the other hand in the year of 2023 we only performed 1 (2%) LC and 5 (10.2%) S with no RC, REC or TC. When comparing colon (RC, REC, LC, TC, S) versus rectal surgery (HAR, LAR, APR, HP) we performed rectal surgery in 43 patients (35.5%) on dV Si versus 45 (37.2%) in dV X/Xi and colon surgery on 6 (5%) dV Si versus 27 (22.3%) dV X/Xi, with a significant difference between implementation period (*p* = 0.002), showing a shift on the type of surgeries performed.

Concomitant to this study, we implemented for all LAR in 2023 and 2024 the RISS, which is the single stapled intracorporeal robotic anastomosis aiming to avoid stapling line intersection and thus reduce fistula rate [14, 15]. The most commonly performed surgery was LAR in 32 (65.3%) x 27 (37.5%) dV Si and dV X/Xi and therefore the RISS was fashioned in 27 (55.1%) x 23 (31.9%) dV Si and dV X/Xi. Other anastomoses utilized are described in Table 2. We had only one conversion to open surgery on dV Si due to bleeding and it was non-significant (*p* = 0.40).

**Table 2:** Operative characteristics stratified by robotic platform (n=121)

Parameter	dV Si (n=49)	dV X/Xi (n=72)	p value
Type of operation ( colon vs rectum)			0.002
Right colectomy (RC)	0	9 (12.5%)	
Right extended colectomy (REC)	0	6 (8.3%)	
Left colectomy (LC)	1 (2%)	0	
Total colectomy (TC)	0	2 (2.8%)	
Sigmoidectomy (S)	5 (10.2%)	10 (13.9%)	
High anterior resection(HAR)	4 (8.2%)	7 (9.7%)	
Low anterior resection (LAR)	32 (65.3%)	27 (37.5%)	
Abdominoperineal resection (APR)	5 (10.2%)	9 (12.5%)	
Hartmann procedure (HP)	2 (4.1%)	2 (2.8%)	
Anastomosis (n= 119)			
RISS	27 (55.1%)	23 (31.9%)	
TTSS	1 (2%)	0	
Double stapled	11 (22.4%)	18 (25%)	
Intersphincteric	2 (4.1%)	3 (4.2%)	
End stoma	7 (14.3%)	11 (15.3%)	
Intracorporeal mechanic	1 (2%)	17 (23.6%)	
Conversion to laparotomy	1 (2%)	0	0.40

* Qui-square

We compared operative times, median (interquartile range [IQR]) on dV Si and dV X/Xi, respectively, on total operative time 320 (190–567) vs. 322.50 (175–680); *p* = 0.74, port placement time 20 (5–85) vs. 20 (5–55); *p* = 0.81, console time 206 (77–373) vs. 240 (45–560) ; *p* = 0.05 and length of hospital stay (LOS) 7 (3–36) vs. 6 (2–26); *p* = 0.12. Total operative time, port placement time and LOS were shorter in dV X/Xi but were not statistically significant. On the other hand, console time was longer in dV X/Xi with borderline statistical evidence. We attribute that to the new type of procedures performed and the adaptation of the team to the new platform. Our operative times are summarized in Table 3.

**Table 3:** Operative times of Si versus X/Xi platform (*n* = 121)

Variable	Si	X/Xi	*p* value
Operative times, Median (IQR)			
Total operative time	320 (190–567)	322.50(175–680)	0.74
Port placement time	20 (5–85)	20 (5–55)	0.81
Console time	206 (77–373)	240 (45–560)	0.05
LOS(days)	7 (3–36)	6 (2–26)	0.12

*Mann-Whitney

LOS -length of stay

IQR - interquartile range

Regarding surgeries performed on the dV X/Xi evaluating colon vs. rectal surgery we found the following Operative times, Median (IQR): total operative time 325 (175–420) vs. 310 (180–680) ; *p* = 0.85, port placement time 19 (5–40) vs. 22(5–55) ; *p* = 0.32, console time 260 (45–370) vs. 230 (96–560); *p* = 0.64 and length of hospital stay (LOS) 6 (2–26) vs. 6 (2–24) ; *p* = 0.39.

Postoperative outcomes were similar between platforms: severe post-operative complications (Clavien Dindo ≥ 3), anastomotic leakage, postoperative ileus, need for NG tube, and 30-day reoperation with no statistical difference. We had no 30-day mortality and regarding the need for parenteral nutrition we had significant differences between platforms (34.7% vs. 16.7%; *p* = 0.02), as shown in Table 4.

**Table 4:** Postoperative parameters Si versus X/Xi platform (n=121)

	Si	X/Xi	p value
Severe complications CD >=3	11 (22.4%)	11 (15.3%)	0.20
Anastomotic leakage	5 (11.9%)	6 (9.8%)	0.75
Postoperative ileus	24 (49%)	32 (44.4%)	0.62
Nasogastric tube	20 (40.8%)	26 (36.1%)	0.60
Parenteral nutrition	17 (34.7%)	12 (16.7%)	0.02
30 day reoperation	6 (12.2%)	5 (6.9%)	0.32
30 day mortality	0	0	

* Qui-square

**Fig. 1:**
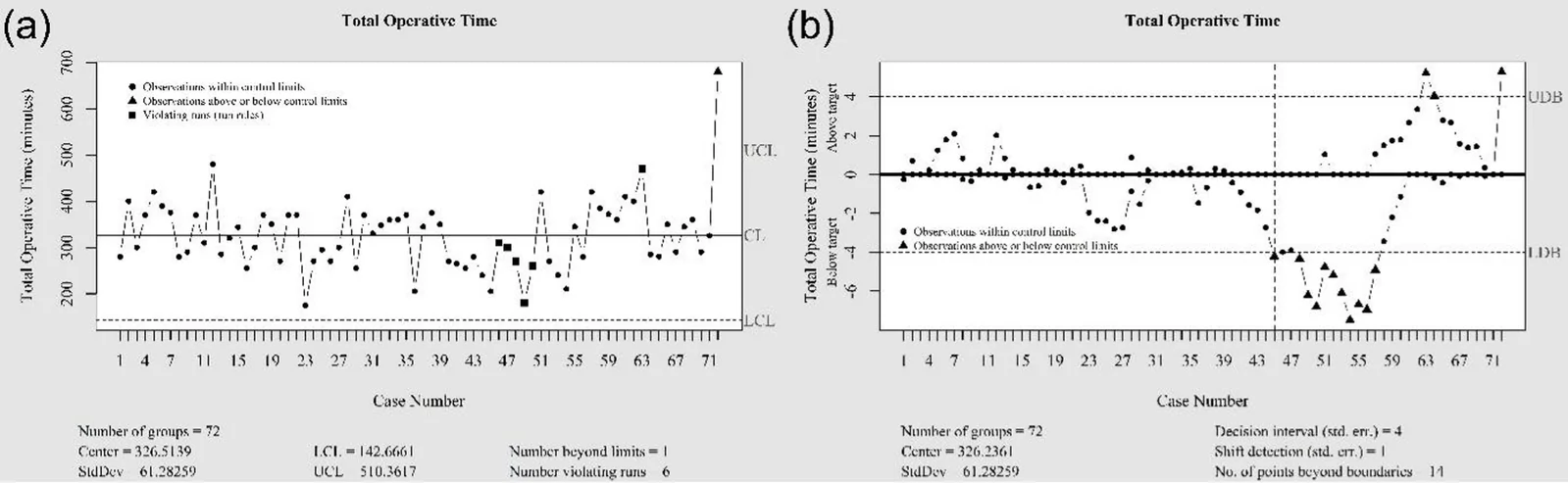
(**a**) Total Operative Time (TOT) against case number. (**b**) Total Operative Time (TOT) CUSUM chart. *UDB* Upper Decision Boundary, *LDB* Lower Decision Boundary, *UCL* Upper Control Limit, *CL* Center Line, *LCL* Lower Control Limit, *StdDev* Standard Deviation, *Std.err.* Standard Error

**Fig. 2:**
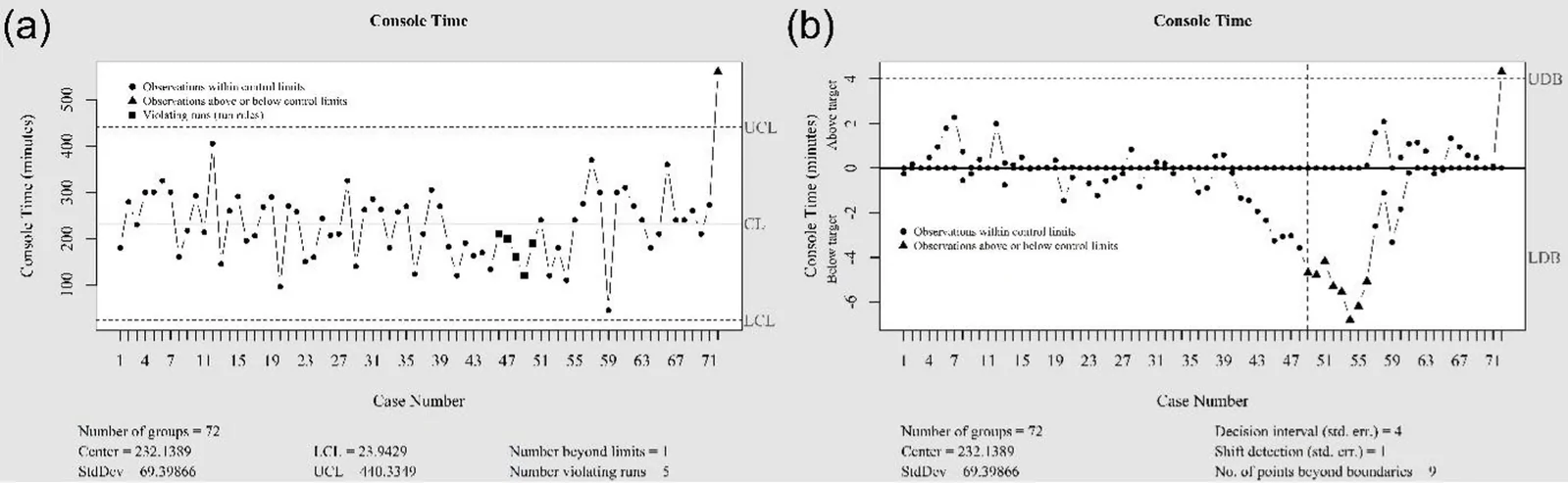
(**a**) Console Time (CT) against case number. (**b**) Console Time (CT) CUSUM chart. *UDB* Upper Decision Boundary, *LDB* Lower Decision Boundary, *UCL* Upper Control Limit, *CL* Center Line, *LCL* Lower Control Limit, *StdDev* Standard Deviation, *Std.err.* Standard Error

We developed a CUSUM curve to evaluate the learning curve on the dV X/Xi platform to determine the number of surgeries needed to achieve proficiency regarding total operative time and console time. The inflection point of the curve represents the transition from the learning phase to proficiency when the value goes under the Lower Decision Boundary (LDB). We demonstrate the dV X/Xi CUSUM chart of total operative time with a learning curve of 45 cases and a console time learning curve of 48 cases. Graphs of raw operative times plotted against case number (a) and CUSUM charts (b) are shown in Figs. 1 and 2 for total operative and console times, respectively.

## Discussion

We conducted a single cancer center cohort of patients submitted to standard colorectal robotic surgeries performed by a team of oncological robotic certified surgeons on a new robotic platform in the same implementation period. Not only surgeons, but the entire surgical team, including nurses, anesthesiologists, and scrub nurses, were habituated with robotic colorectal surgeries.

It is well established that robotic surgery allowed for a series of improvements in colorectal surgery, and since the dawn of robotic surgery, it has evolved over the years aggregating new features for safer and more precise surgery with better cost- effectiveness. It is reported that the da Vinci Xi platform, with its slimmer boom-mounted arms, extended instrument reach, guided targeting and simplified single docking, allows improved multiquadrant access compared to the Si platform, as described by Protyniak et al. [22].

When comparing the different implementation periods, we noticed a significant increase in procedure diversity in the latest period: we performed 17 (23.6%) colectomies on dV Xi which were not performed on dV Si. With the dV Xi we used a single docking template on the left flank for any procedure and a simple boom shift on quadrant change, allowing even total colectomies to be performed.

Regarding the differences in the use of the new robotic platform, a patternized linear port configuration was established. Other improvements include a lighter 8 mm camera with better imaging resolution, easier shift on the 30° endoscope from 30° up to 30° down on a simple screen command. Less arm collision: flex joints with only 1 fist width needed.

As current literature states, robotic surgery is primordial in low anterior resections as shown in the REAL Trial conducted by Feng Q. et al. resulting in statistically significant longer disease-free survival and less locoregional recurrence as well as better urinary and sexual function. This reveals better oncological long-term results and not only lower conversion rates on subgroup analysis as the ROLARR trial conducted by Jayne et al. had previously suggested [6, 7].

On the other hand, further studies are warranted to better understand the potential benefits of robotic colectomy compared with laparoscopic surgery. Many authors have questioned the use of robots in hemicolectomies due to its higher cost and longer operative time. Some authors discussed benefits such as easier intracorporeal bowel anastomosis under vision with less mesenterial traction, freedom of site extraction and that it might facilitate complete mesocolic excision with central vascular ligation [23–25].

Robotic platform transition requires adjusting the operating room (OR) setup, establishing new port configurations and docking protocols, and ensuring the surgical assistant’s familiarization. Most importantly, it demands attending surgeon proficiency. To assess this latter parameter, we utilized CUSUM analysis to determine the surgeon’s learning curve on the new robotic system.

When mastering a new platform, the continuous repetition of tasks will allow the surgeon to improve his or her performance, which will translate into an initial decrease in operating time. This period is called the “learning curve”. After this initial decrease in operating and console time, usually we have a new increase due to the improved surgical confidence leading to more challenging cases. The CUSUM method allows us to statistically represent the learning curve phenomena showing operative time variation in consecutive procedures [19–21].

Our CUSUM chart showed a dV X/Xi learning curve of 45 and 48 cases for total operative time and console time respectively. We consider it to be a high number of cases when compared to current literature. Chun-Yu Lin, et al. learning curve was 31 cases for colon and rectal robotic surgeries performed by two surgeons, in Yu-Kang Tseng, et al. retrospective analysis for a single surgeon found that 16 cases were enough to achieve the plateau [26, 27].

With the increase in colectomies performed, we expected a shorter console time, considering it a less complex surgery when compared to rectal surgery. But even so, we had increased console time. Some factors may have contributed to a longer learning curve in our study, such as the diversity of procedures performed and no previous simulation or proctorship in the implementation of the new platform. Postoperative outcomes were maintained and the only statistical difference was the need for parenteral nutrition, more frequent in the dV Si (34.7% vs. 16.7%; *p* = 0.02). The higher frequency of parenteral nutrition observed during the Si period may have been influenced by differences in postoperative gastrointestinal recovery, case complexity, nutritional practices. Indications for parenteral nutrition were not evaluated in detail, so this finding should be considered exploratory.

Based on our experience, implementation of a new robotic platform should be supported by a structured transition program, including platform-specific didactic training, simulation-based practice, and supervised initial cases. Case complexity could be progressively increased as proficiency is achieved, while objective performance monitoring, including operative metrics and learning-curve assessment, could help identify the adaptation phase and ensure safe implementation.

Our aim was to describe our experience with the transition between robotic platforms, including both its advantages and shortcomings. The limitations of our study include the heterogeneity of procedures between the two groups, which reflects the complexity of real-world clinical practice. In addition, variations in anastomotic techniques and the introduction of a novel technique during the same period may have influenced the observed outcomes.

Other limitations of our study were the lack of hands-on training before utilizing the novel platform. Robotic instruments availability could also have negatively impacted the learning curve since we don’t routinely use robotic staplers, clip appliers or advanced bipolar energy instruments due to their cost. We also have to take into consideration that using operative time as a marker for learning has its critics since the learning process involves a surgeon’s technical capability and the rapport of the entire surgical team.

These limitations are inherent to real-world observational studies. Our findings suggest that robotic platform transition can be implemented safely without compromising short-term outcomes, although a period of adaptation may occur. Rather than being considered solely a technological upgrade, platform transition may benefit from a structured implementation process, incorporating didactic training, simulation, supervised initial cases, and objective performance monitoring which may facilitate adaptation and minimize the impact of the learning curve. Further studies are warranted to validate these findings and define optimal strategies for robotic platform transitions.

## Conclusion

We described our institutional experience during the transition between robotic platforms. The transition was not associated with significant changes in most postoperative outcomes, although it was associated with a longer console time. Our findings suggest that previous robotic experience alone may be insufficient for a seamless platform transition and structured retraining should be advised.

## Data Availability

No datasets were generated or analysed during the current study.
